# An Indoor Navigation Methodology for Mobile Devices by Integrating Augmented Reality and Semantic Web

**DOI:** 10.3390/s21165435

**Published:** 2021-08-12

**Authors:** Jesus Ivan Rubio-Sandoval, Jose L. Martinez-Rodriguez, Ivan Lopez-Arevalo, Ana B. Rios-Alvarado, Adolfo Josue Rodriguez-Rodriguez, David Tomas Vargas-Requena

**Affiliations:** 1 Reynosa Rodhe Multidisciplinary Academic Unit, Autonomous University of Tamaulipas, Reynosa 88779, Mexico; a2133720100@alumnos.uat.edu.mx (J.I.R.-S.); arodriguez@docentes.uat.edu.mx (A.J.R.-R.); dvargas@docentes.uat.edu.mx (D.T.V.-R.); 2 Centro de Investigación y de Estudios Avanzados del I.P.N. Unidad Tamaulipas (Cinvestav Tamaulipas), Victoria 87130, Mexico; ilopez@cinvestav.mx; 3 Faculty of Engineering and Science, Autonomous University of Tamaulipas, Victoria 87000, Mexico; arios@docentes.uat.edu.mx

**Keywords:** indoor navigation, augmented reality, Semantic Web

## Abstract

Indoor navigation systems incorporating augmented reality allow users to locate places within buildings and acquire more knowledge about their environment. However, although diverse works have been introduced with varied technologies, infrastructure, and functionalities, a standardization of the procedures for elaborating these systems has not been reached. Moreover, while systems usually handle contextual information of places in proprietary formats, a platform-independent model is desirable, which would encourage its access, updating, and management. This paper proposes a methodology for developing indoor navigation systems based on the integration of Augmented Reality and Semantic Web technologies to present navigation instructions and contextual information about the environment. It comprises four modules to define a spatial model, data management (supported by an ontology), positioning and navigation, and content visualization. A mobile application system was developed for testing the proposal in academic environments, modeling the structure, routes, and places of two buildings from independent institutions. The experiments cover distinct navigation tasks by participants in both scenarios, recording data such as navigation time, position tracking, system functionality, feedback (answering a survey), and a navigation comparison when the system is not used. The results demonstrate the system’s feasibility, where the participants show a positive interest in its functionalities.

## 1. Introduction

In recent years, the importance of navigation systems has been increasing due to their accessibility and functionality, allowing users to access from their mobile device to information and location of places anywhere in distinct scenarios. The navigation systems are categorized as *outdoor* and *indoor*. Outdoor navigation systems use Global Positioning System (GPS), public maps, or satellite images. However, such data are unsuitable for indoor navigation systems operating inside buildings or small semi-open areas, such as schools, hospitals, and shopping malls. Due to the incompatibility and low accuracy of the standardized technology of such systems [1], the development of indoor navigation systems has been the subject of diverse research works [2,3,4,5], resulting in a variety of technologies, methods, and infrastructure for its implementation, being difficult to establish a standard for the development of these systems. The implementation of indoor navigation systems is highly beneficial for locating and extracting information about points of interest within buildings, especially when users are visiting the location for the first time, the indoor signs are not very clear, or there is no staff available to provide directions.

Most of the works for navigation focus on the development of positioning methods, based on wireless networks (IR, sensors, ultrasound, WLANs, UWB, Bluetooth, RFID) [2,4,6,7,8]; Augmented Reality (AR), but not providing contextual information [3,9,10,11]; or the development of methods providing contextual information regarding the environment, but without offering indoor navigation [5,12,13,14]. Thus, such works require a suitable infrastructure for indoor position tracking and to adapt the system to specific environments. In recent years, Augmented Reality [3,15] and Semantic Web [16] technologies have grown in relevance, separately, as enrichment means for the development of navigation systems. These technologies provide the user with more interactive navigation experiences and enhancing information consumption and presentation. Although AR offers capabilities for tracking the user’s location and presenting augmented content, the Semantic Web offers capabilities for querying, sharing, and linking public information available on the Web through a platform-independent model. Combining both technologies for indoor navigation would enhance the user experience, linking available indoor place information to navigation paths, navigation environment, and points of interest. However, according to our knowledge, there are no works exploiting AR and the Semantic Web for developing indoor navigation systems.

Therefore, this paper presents a methodology for developing an indoor navigation system based on AR and the Semantic Web. These combined technologies allow displaying navigation content and environment information to resemble the functionality provided by outdoor navigation systems without additional infrastructure for its implementation. The proposed methodology consists of four modules that communicate with each other, establishing alignments for spatial representation both real and virtual, the structure of contextual information, the generation of routes, and the presentation of augmented content to reach a destination point.

Academic environments are suitable for navigation systems due to the high number of external visitors and public information availability. Thus, the proposed methodology was tested by implementing a mobile device system prototype related to the academic context, where the points of interest refer to offices, laboratories, and classrooms. In particular, two buildings (from independent institutions) were modeled through a graph space model (routes, places) with points of interest distributed across different floors. Moreover, it was defined and implemented an ontology that supports a knowledge base (KB) with information of the academic or administrative staff (associated with the points of interest). Such KB is helpful to disseminate information through the Web and provides contextual information as virtual objects during navigation. Both scenarios were evaluated using different navigation tasks and participants.

The contributions of this paper can be summarized as follows:

- The design of a methodology for indoor navigation systems incorporating Augmented Reality and Semantic Web technologies.
- The implementation and availability of a functional indoor navigation prototype application and ontology for academic environments.

This paper is organized as follows. Section 2 describes the related work regarding the development of indoor navigation systems using AR and Semantic Web technologies. Section 3 describes the proposed methodology and its modules. Next, in Section 4, the implementation process is presented. In Section 5, the experiments and results are described, stating the evaluation criteria and study cases. Afterward, Section 6 presents a discussion over the prototype’s performance; and finally, Section 7 states the conclusions and further work.

## 2. Related Work

Diverse works have been proposed for performing indoor navigation incorporating AR, where the methods, techniques, and technologies commonly used for their development are mainly composed of four stages: a representation of the building where the system will be used, a method for the positioning/tracking of user movements, the application of pathfinding algorithms, and the presentation of navigation content through AR indications [17,18,19]. The following subsections present some works for indoor navigation, incorporating AR, and a discussion of the most common techniques for such a purpose.

### 2.1. Indoor Navigation Using AR

Existing works usually address the four stages mentioned before with a particular focus on positioning and navigation. Kumar et al. [15] use the camera and inertial sensors from an AR framework to track 3D point clouds, which represent locations to aid the navigation inside a fully 3D spatial model (previously scanned). Then, they store anchors in a cloud database and computing the shortest route through the A* algorithm.

Although not using AR for position tracking, Cankaya et al. [20] use such a technology for navigation guidance through virtual arrows. They track the user position using the device’s accelerometer, showing the navigation route by selecting both the start and destination points, and calculating the shortest path using Dijkstra’s algorithm on a node-based spatial model.

Gang et al. [3] propose a hybrid location technology that combines Bluetooth beacons and geomagnetic field and mobile device motion sensors within digital 3D spaces. They calculate the navigation route using the Dijkstra algorithm and display navigation guidance through AR or visualizing the created 3D model. Similarly, Huang et al. [21] use Lbeacons (Bluetooth low energy beacons) for positioning and Dijkstra for pathfinding.

Matuszka et al. [16] focused on the integration of AR and Semantic Web technologies to develop navigation systems proposing an architecture that uses AR to display virtual arrows with the aid of QR codes on the floor and the Semantic Web for constructing the routes without giving contextual information about the environment. The tracking location function is done by measuring the movements through the phone’s inertial sensors, relating the position using QR codes and a third app map provider.

Although these previous works offer positioning and navigation capabilities, they do not offer contextual information about the environment (descriptions about locations). In this regard, Kokorogianni et al. [12] developed an application that uses AR for the visualization of information contained in a database related to a school institution, providing information about office schedules and activities to students and visitors, but lacking navigation functionalities. Similarly, Contreras et al. [22] offers academic information to students and visitors of a university through AR, storing and querying the information through an ontology that links different entities, allowing complex searches. However, it does not offer navigation methods towards points of interest. Thus, to solve the lack of navigation functionalities (and information consumption), Delail et al. [23] proposes a mobile system for indoor navigation that uses AR to display information of points of interest stored in a database (during navigation activities). The user position is obtained by detecting markers placed in the environment, and it manages its tracking through the mobile device’s sensors. They use a computer-designed plane to present a spatial model, overlaying a third-party map system that uses latitude and longitude as coordinates. Moreover, the navigation route is obtained through the A* algorithm and displayed as a drawn line on a map.

Although not using a mobile device, Chidsin et al. [24] employ an RGB-D camera for data collecting, mapping, and positioning, where the navigation is supported by the position of destination in a point cloud map and Simultaneous Localization and Mapping (SLAM) algorithms, displaying the location name during navigation.

### 2.2. Common Component Strategies and Tools

As can be seen, the works described above perform indoor navigation with AR under diverse conditions and tools for each of the four stages previously mentioned. Regarding the representation of the building, techniques such as graphs, point clouds, and 3D models are often used. However, selecting between one or another modeling technique usually depends on the strategy for rendering the building of interest, and creating a map or drawing, for example, by capturing plans to create a graph [19] or those that capture a representation by traversing locations and generate 3D models or point clouds [24]. On the other hand, regarding data models, proprietary formats are often used (and adapted to the application’s requirements) to represent building interiors, routes available to the user (there may be restricted areas), and points of interest information (regularly with relational databases). It would be desirable and useful to incorporating formats for data integration and platform independence, such as information represented with Semantic Web standards [25,26].

Regardless of the model, there must be a way to position the device. In this sense, different strategies are often used, such as the incorporation of external elements (beacons, WiFi networks), use of sensors (accelerometer, compass, gyroscope), and use of computer vision (to detect features in images that help detect the position and movement of the user). Each technique has its opportunities and difficulties. Although marker reading offers accurate positioning, it also involves constant interaction by the user; Bluetooth and WiFi-based systems support user tracking, but these devices are not always available; computer vision-based systems offer some independence from external sensors but tend to have high energy consumption [24].

The last part of the process corresponds to selecting a navigation route between two points and displaying the result by means of AR. Approaches usually obtain the navigation path by traditional pathfinding algorithms such as Dijsktra or A*, but have also resorted to stored information with possible directions [6] or by SLAM algorithms [24]. On the other hand, the visualization of navigation directions by means of AR is usually through virtual objects such as arrows, lines, or animated objects [27].

The aforementioned systems and approaches have been tested on a diversity of areas and buildings. For example in hospitals [21], shopping mall [16,19], universities/college [6,27], and so on. A summary of the components of the related work can be seen in Table 1.

Even though the works present diverse utilities and benefits for indoor navigation, it is desirable to have a way of exchanging diverse components per stage, either for positioning, navigation, and so on. In addition, most of the works do not provide information about the environment beyond navigation; that is, they do not provide contextual information to support the enrichment of the environment. Moreover, information on the points of interest is usually stored/retrieved with relational or ad-hoc data models that limit its interchangeability with other applications or services. This last remark provides an opportunity to integrate the Semantic Web in indoor navigation systems to structure and consume points of interest information.

## 3. Methodology Design

**Figure 1:**
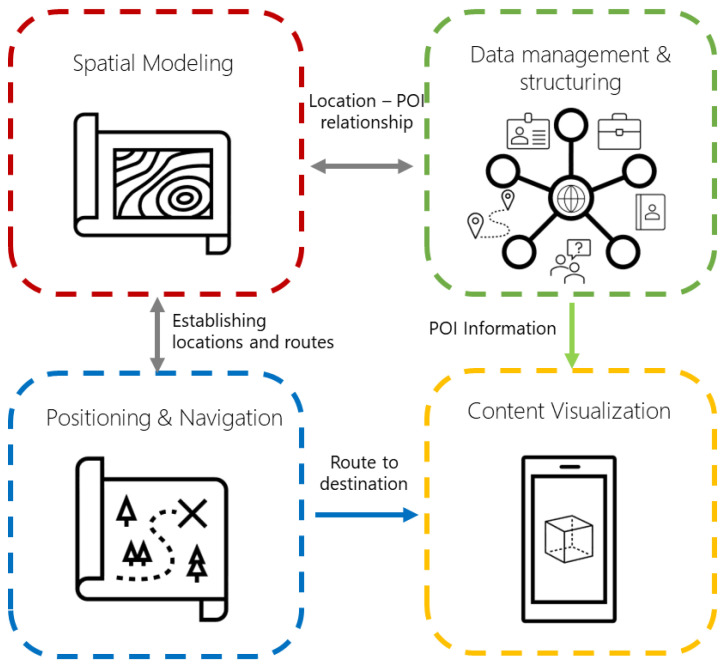
Methodology for developing indoor navigation systems based on AR and Semantic Web.

This section presents the proposed methodology for the development of indoor navigation systems for mobile devices. It integrates AR and Semantic Web technologies as methods for navigation and contextual information consumption. According to the components stated in the related work for the development of indoor navigation systems, this proposal is based on four modules presented in a sequential order in which the methodology is performed: (a) *Spatial modeling*, for representing places, points of interest, and routes; (b) *Data management and structuring*, for contextual data representation and consumption; (c) *Positioning and navigation*, for user position tracking and pathfinding; and (d) *Content Visualization*, for displaying navigation routes and point of interest information through AR elements. Figure 1 summarizes the modules of the proposed architecture. Although the following subsections present general design descriptions of the modules in the methodology (without focusing on specific programming tools), the next section presents particular details (strategies, tools, recommendations) for a system implementation following such modules.

### 3.1. Spatial Modeling

The objective of this module is to create a spatial model for the computational representation of a building. Thus, the model uses a coordinate system for the location of points of interest that will help in the generation of navigation routes for guiding the user. This coordinate system requires three axes to represent a multistory building and for the degree of orientation of the mobile device to present the virtual content concerning the user’s position and orientation.

In other words, a spatial model m∈M is composed of a set of points $P=\{p_{1},p_{2},\ldots ,p_{i}\}$ and a subset of points of interest POI⊆P. In this regard, a point takes the form p=(x,y,z)∈X×Y×Z, where X,Y,Z define the sets of valid coordinates for the modeled scenario. Additionally, it can be applied a rotation over the model, keeping the *y* axis fixed.

### 3.2. Data Management and Structuring

This module aims to create a Semantic Web knowledge base (KB) that stores information about the points of interest from the spatial model. The KB is structured and supported by an ontology that models the navigation environment and the information presented to the user at navigation time. In this way, the data consumption through the KB is helpful for the application due to its well-defined structure, flexibility, and association with real-world entities (for more intuitive queries). According to the Semantic Web guidelines, the ontology must represent all domain of interest concepts in a clear and well-defined form. Moreover, this ontology must reuse existing ontologies (or accepted vocabularies), such as iLOC ontology [26], ifcOWL [29], FOAF [30], DBpedia [31], to mention a few. Then, a new ontology can be extended and adapted to the indoor navigation environment at hand. Therefore, the entities of the navigation environment, points of interest, spatial model, and any other contextual information can be represented in the KB.

For practical reasons, most of the KBs are structured and represented according to the *Resource Description Framework* (RDF) [32] as a directed edge-labeled graph [33]. Formally, RDF is a data model (whose main component is the RDF triple) that defines elements such as *Internationalized Resource Identifiers* (IRIs) [34], to identify resources globally on the Web; *blank nodes*, particular components used for grouping data (acting as parent nodes); and *literals*, representing strings or datatype values. Thus, given infinite sets of IRI elements *I*, blank nodes *B*, and literals *L*, an RDF triple takes the form t=(s,ρ,o)∈(I∪B)×I×(I∪B∪L). For example, entities as *s* are expected to be uniquely associated with their corresponding p∈POI by means of some property ρ. Then, other properties in the ontology are helpful to define additional information about *s* and enrich the points in the spatial model (and used as contextual information during navigation). Please note that the KB is queried at navigation time for presenting information to the user about the indoor navigation environment (at the beginning), contextual information during route traversing, and points of interest arrival (virtual landmarks).

### 3.3. Positioning and Navigation

After modeling the coordinate system and the KB with the POI information, this module aims to obtain the user position within the spatial model and the navigation through an obtained route. In this sense, it is required a positioning method that tracks the user movements in two phases:

- *Establishing the initial location.* This phase establishes the user’s initial position within the spatial model, presenting the whole navigation environment and virtual objects relative to that position. Thus, an initial position can be defined as $p_{s}\in P$, which can be established in multiple ways, as previously mentioned. For example, through marker detection [15], Lbeacons and sensors [21], and WiFi fingerprinting [27], to mention a few.
- *Tracking user’s movements.* Once the initial position is established, the user’s position is tracked and updated to correctly place all virtual objects involved during navigation (displayed in positions relative to the tracked movements). Thus, when the user is moving throughout the building, its position is commonly updated from a point to an adjacent point $p\to p^{\prime }$ through the mobile device’s sensors (e.g., WiFi, Bluetooth, compass) [21,22,23] or computer vision [24,28], to mention a few.

A positioning and tracking strategy is later defined (Section 4) using the mobile device’s motion sensors and visual features in the environment. On the other hand, the navigation function is aimed at helping users reach a point of interest from a starting position. A pathfinding algorithm is applied to the spatial model to create the shortest navigation route between the pair of points. In other words, given the function $f\left(p_{s},p_{o},m\right)=\left\{p_{s},\ldots ,p_{o}\right\}$, where $p_{s}$ is the initial position, $p_{o}$ a point of interest, and *m* a spatial model, it returns a succession of points in *m* to travel from $p_{s}$ to $p_{o}$. For example, if the spatial model is represented by a graph [20,22,28], it is possible to apply a pathfinding algorithm [35] such as A* or Dijkstra.

### 3.4. Content Visualization

This final module aims to present the information regarding the points of interest stored in the KB and the visualization of navigation routes through virtual objects (using AR and the components above described), taking into account the best way to present such content without affecting its consumption. In this sense, two components are proposed:

- *Data presentation*. This component presents the information of the points of interest through non-augmented interfaces. The goal is to show an interface to read and view concisely such information. Moreover, it allows the user to begin the $p_{s}$ setting and select $p_{o}$ for navigation.
- *Navigation guidance.* In contrast, navigation guidance is provided by *augmented objects* visible through the mobile device screen, ensuring that the user can follow the guidelines while still paying attention to their step. Of course, according to the user position tracking, such augmented objects stay fixed at the points given by $f(p_{s},p_{o},m)$ so that the user can follow the navigation route. Moreover, additional augmented objects can be displayed during navigation, such as discovering point of interest within a radio from the user position (contextual information) or when the desired position is reached.

## 4. Methodology Implementation

This section presents a prototype implementation (as a mobile device system) based on the proposed methodology. This indoor navigation system aims to support users in finding an office, laboratory, classroom, or finding a place according to the staff member (e.g., professor *x* assigned to office *y*). Although it is possible to implement the proposed methodology using different tools, the prototype was developed with Unity 3D (it is a development engine that is compatible with AR frameworks and handles coordinate systems to place virtual objects) and designed for scenarios related to academic spaces. Thus, the ontology and navigation paths were designed to cover the needs of students and guests of such spaces. However, although this proposed implementation can be reused for modeling other types of buildings (such as hospitals, shopping malls, museums), the spatial modeling and the KB must be developed and adapted to the scenario and data. Details of the implementation for each module of the methodology are next described. The project’s repository is available online (https://github.com/RepoRodhe, accessed on 7 June 2021).

### 4.1. Spatial Model

To specify a relationship in the data, the spatial model is represented with a graph with several nodes and points of interest distributed over different building floors. The process is as follows:

- *Digitalization*. Scale plans of the building are used to create the graph, generating digital images used as a reference for placing the graph’s nodes and route visualization being imported into Unity3D. Thus, the plans should be digitized, and their scale (pixels per unit) must be defined to import them. In this sense, 1 unit in Unity3D represents approximately 1 m in a real environment (digitalized plans and scale examples are later presented in the experiments section). Moreover, to keep the images homogeneous (no annotations of measurements, indications, etc.), it is recommended to import the plans using a design software tool such as SketchUp [36].
- *Graph creation.* Within the system, nodes refer to points of interest or landmarks within buildings, storing information that aids in the extraction of information from the KB (ready for Semantic Web usage). On the other hand, edges represent corridors and help in the creation and visualization of navigation paths. In this regard, native Unity3D objects were used to represent the graph nodes and edges. Thus, the nodes have their corresponding location within the native coordinate system of the engine using three-axis to enable multi-floor positioning. In turn, each of the nodes contains an array of adjacent nodes (a GameObject array in C#), which has the function of representing the edges of the graph. Although the methodology does not limit the representation of the number of floors of a building, the implemented prototype uses a spatial model of two floors for the two tested scenarios (later presented in the experiments). It is worth mentioning that the performance of the Unity3D platform is affected according to those objects that are visible at any given time [37] and thus, it is possible to have several objects for modeling any building.

### 4.2. Data Structuring

**Figure 2:**
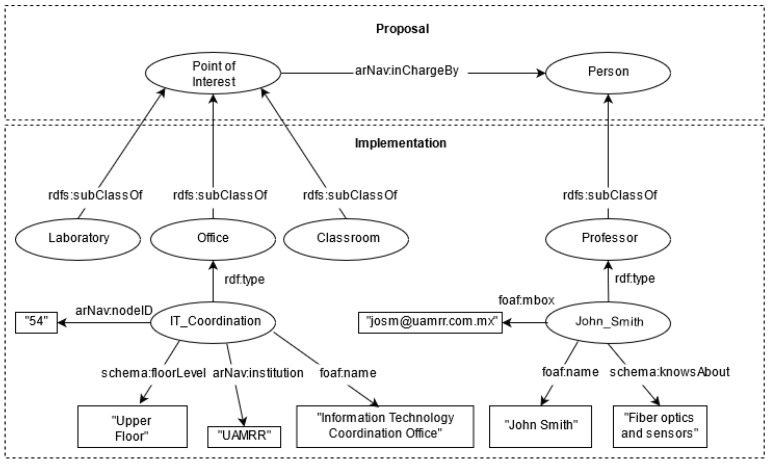
Designed ontology for contextual information in the academic domain.

For the ontology construction and implementation, a hierarchy of concepts was designed to store helpful information for the user concerning the points of interest. In this sense, it was defined an ontology for describing points of interest information, which is based (and aligned) on the iLOC ontology [26] (for publishing indoor locations and navigation data). However, since the prototype system domain is part of the academic context, the ontology is enriched with classes representing offices, laboratories, and classrooms, storing information about the teaching staff associated with each location, their activities, academic specialties, and contact information. Particular details for the construction, feeding, and consumption of the KB (supported by the ontology) are as follows:

- The Protégé tool [38] was used to build the ontology, maintaining a hierarchy of classes to reduce redundant properties and in compliance with the Linked Data principles [39]. The scheme of the proposed and implemented ontology can be seen in Figure 2. In this sense, arNav refers to the prefix of the application, Proposal refers to the smallest part of the ontology to work under any environment, and Implementation gives particular definitions according to the domain at hand, in this case for the academic domain. Please note that some labels and properties (*alignments, equivalences*) are not shown to keep the figure legible.
- Once the ontology was defined, the information for creating the KB can be manually entered (RDF files) or through a system assistant. For the implemented system, the applied strategy was to load information from a spreadsheet file into the ontology using the Protégé tool Cellfie (https://github.com/protegeproject/cellfie-plugin, accessed on 7 June 2021), which allows the creation of classes, entities, attributes, and relationships through the specification of rules. Please note that information from multiple navigation scenarios can be stored in the same KB. In this sense, the KB for the two cases in the experiments contains 1347 RDF triples (81 points of interest from a total of 149 entities).
- The client-server model was used to keep the KB updated and reduce the mobile device’s processing load regarding information consumption. For this purpose, the Virtuoso Open Server tool [40] was used since it supports RDF/OWL storage and offers a SPARQL endpoint for its consumption. For testing purposes, the developed prototype was configured under a local network using a 2.4 Ghz WiFi connection, where the SPARQL queries were submitted from the mobile device to the server through HTTP requests using Unity3D’s native networking library and then parsing the JSON response for the correct visualization in the prototype interface. Five different SPARQL queries are performed through the menus and interfaces offered by the prototype, extracting the following information: (1) Information linking the nodes with the points of interest of the navigation environment, (2) Information of all points of interest by category, (3) Information of all academic staff, (4) Detailed information of a selected point of interest, and (5) Detailed information of a person. Listing 1 presents a SPARQL query example to retrieve all the information of a point of interest of a certain institution and the staff members related to that location. The retrieved information by such query is later used by the next module for visualizing content.

Listing 1. **SPARQL query example.**

**PREFIX** arNav: <http://www.indoornav.com/arNav#>
**PREFIX** foaf: <http://xmlns.com/foaf/0.1/>
**SELECT** ∗ **WHERE** {
              arNav:Research_Lab arNav:nodeID ?id;
                  foaf:name ?name;
                  sch:floorLevel ?floor;
                  arNav:institution "UAT".
              OPTIONAL{
                  arNav:Research_Lab arNav:inChargeBy ?person.
                  ?person foaf:firstName ?firstName;
                      arNav:fatherName ?lastName;
                      arNav:extension ?extension.
                  OPTIONAL {?person foaf:title ?nameTitle.}
                      }
              OPTIONAL{
                  arNav:Research_Lab sch:description ?desc.
                  }
              OPTIONAL{
                  arNav:Research_Lab arNav:areas ?areas.
                  }
          }

### 4.3. Positioning and Navigation

For the implementation of the positioning function, the AR Foundation framework was used [41], offered by the Unity3D engine. It integrates functionalities from other AR frameworks on the market, such as Google’s ARCore [42] and Apple’s ARKit [43], so a mobile device compatible with either of the two frameworks, described on their respective website, is required to use the AR functions with the AR Foundation framework. Thus, the positioning and navigation are addressed as follows:

- Initial position. In order to establish the user’s initial position within the spatial model, QR codes were used to store information about the building and the location under the Unity3D coordinate system. The ZXing library [44] was used for decoding the QR codes. It is worth mentioning that a QR code must be scanned if the user position is lost (recalibration/repositioning), resuming the navigation automatically.
- Tracking. Once the initial position is established, the user location is tracked down using the pose tracking component (TrackedPoseDriver) from the Unity Engine Spatial Tracking library and attaching it to the AR Camera. This component captures visually distinctive features in the environment to work as anchors and track the device movements and rotations relative to the detected features’ position. Such component is also supported by the mobile phone’s sensors (gyroscope and accelerometer). Afterward, the detected movement changes are processed by the ARSessionOrigin component, provided by the AR Foundation framework, to translate the movements to the Unity Engine coordinate system.
- Navigation. Regarding navigation, the graph denoting the spatial model is represented as an adjacency matrix (using each node’s adjacency nodes array), so then the Dijkstra’s pathfinding algorithm [35] is applied for finding the shortest route for navigation towards the destination.

**Figure 3:**
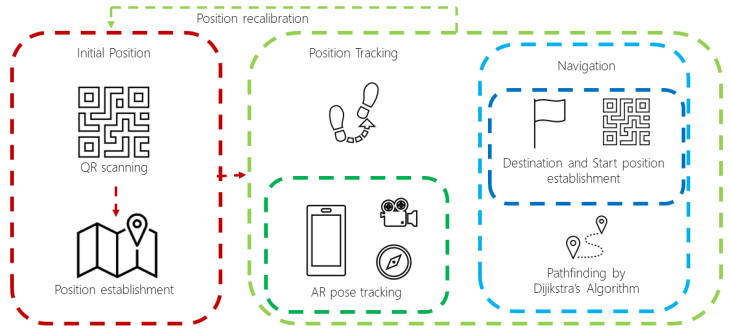
Implementation diagram of the Positioning and Navigation component.

The interaction of the components in the positioning and navigation implementation is summarized in Figure 3.

### 4.4. Visualizing Content

Taking advantage of the AR framework capabilities to display content, the user can visualize the virtual navigation guidance and the points of interest information stored in the KB through different interfaces.

**Figure 4:**
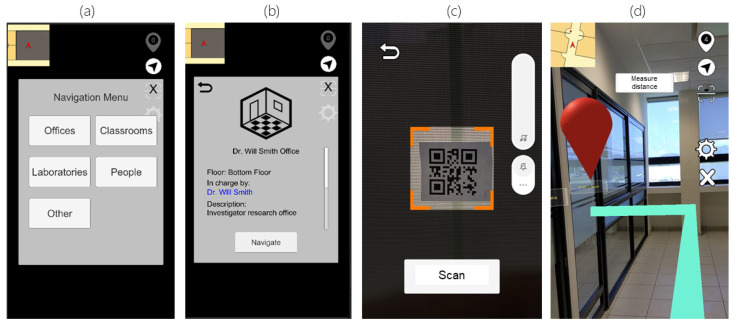
Developed application’s interfaces examples. (**a**) POI types menu, (**b**) POI contextual information, (**c**) QR scanning interface, (**d**) AR line for path guidance.

The visualization starts by scanning a QR code, which helps load the corresponding data for the building where the user is located. Afterward, it is displayed a menu categorizing the entries in the ontology regarding the stored classes (laboratories, offices, and classrooms). At this step, the user can select a point of interest to visualize the stored information and set its location as the destination to start the navigation. This action prompts the user to scan a QR code to establish the initial location, calculating the shortest route between the two locations (using the Dijkstra algorithm). The generated route is displayed by an augmented virtual line (LineRenderer) that connects all the nodes involved in the route and can be viewed through the mobile device’s screen. An example of all the different interfaces implemented is displayed in Figure 4.

## 5. Experiments and Results

This section presents the experiments and results of the implemented prototype under two scenarios related to academic buildings. The idea is to test the performance of the prototype regarding navigation time, accuracy, and usability. Along these lines, a description of the general evaluation criteria is first presented, and then particular details and features for each case are described.

### 5.1. Evaluation Criteria

The performance of the components in the prototype is evaluated regarding the following criteria:

- *Position setting*. The effectiveness of QR code scanning is measured. The initial positioning method considers how often this function was performed correctly, positioning the user in the right place and orientation.
- *Distance offset*. The efficiency during continuous tracking of the user’s location using the AR functionality by measuring the distance offset between the user’s real location and the one registered by the system.
- *Navigation time*. This aspect considers the navigation time in seconds to the different points of interest established as objectives for the tests. Additionally, it is compared the time recorded in the scenarios where the mobile device is used with the proposed system and in those where the user navigates without using it.
- *AR display issues.* This aspect counts the number of errors during the content display presented for virtual objects, particularly the incorrect display of navigation guides and menus, preventing partial or incomplete navigation.
- *User feedback*. The degree of user satisfaction is evaluated regarding the system usability and the contextual information by applying a survey based on a 5-point Likert scale [45] and the user agreement degree by the Fleiss kappa measure [46].

Since the case studies were tested by adapting such criteria, the following section present the particular features used for each case.

### 5.2. Case 1: University Building

The first case of study for the implemented system was prepared for a building of the Autonomous University of Tamaulipas, Mexico (https://rodhe.uat.edu.mx/inicio/, accessed on 7 June 2021). The mobile device used to conduct the tests was a Samsung Galaxy S7 running Android 8.0. The data were collected through a log script. Additional details of the scenario and participants of the study are presented in the following subsections.

#### 5.2.1. Participants

For this navigation environment, 24 undergraduate students (from the University) with different scholar grade levels and areas were invited to perform navigation tests. Some additional features of the users are as follows:

- All users are familiar with the mobile platform and understand the usefulness and scope of augmented reality applications.
- 9 of the 24 participants had no prior knowledge of the environment (being unfamiliar with the location of the targets) and the remaining ones only have knowledge of some areas. However, all the participants were instructed and supervised to only follow the directions provided by the implemented system.
- Each of the participants performed a set of 6 navigation tasks, half of them using the implemented system and the rest without it. Moreover, each of the users alternated the tasks where the implemented system was used, generating a data set of 144 entries in total (72 entries using the implemented system and 72 without its use).

#### 5.2.2. Scenario Description and Navigation Tasks

**Figure 5:**
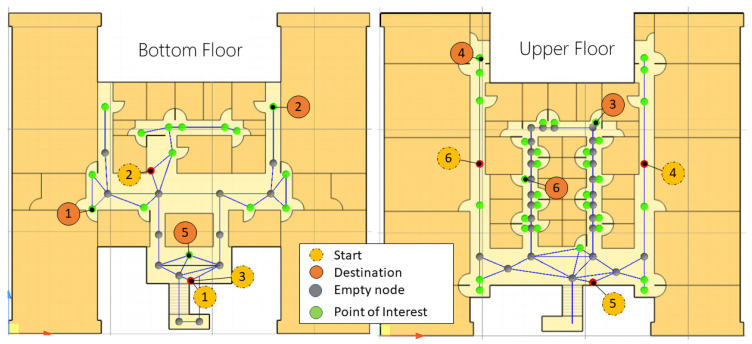
Scenario and navigation tasks for the University building.

The building is composed of two floors. After digitizing the building plans, images of 662 × 662 pixels were generated for each floor, representing an area of 679 m^2^. Then, such images were imported into Unity3D under a scale of 18.64 pixels per unit. Regarding the spatial model, 93 nodes were defined, from which 45 refer to points of interest accessible to users. The navigation scenario for this case is depicted in Figure 5. Please note that nodes and points of interest are described together with six testing routes denoted by *Start* and *Destination* points (a QR code is located on every *Start* point). Thus, the participants are intended to navigate within the scenario between such points. Table 2 shows in detail the six navigation tasks designed for this environment.

#### 5.2.3. Results

This section presents the results obtained from the performed navigation tasks, interpreting the data and events that occurred during the experiments. Each navigation task was completed 24 times by the participants (12 with the implemented system and the rest without it). Moreover, on average, the time to complete the tasks was 46.25 s, with a traveled distance of 152.30 m per participant. The results for the assessed criteria are described as follows.

##### Position Setting

Taking into account the 72 cases where the prototype was used, a total of 94 QR code scans were performed to locate the user within the device’s coordinate system (22 for repositioning), positioning the user correctly on 91 occasions (96.8% accuracy).

##### Distance Offset

**Figure 6:**
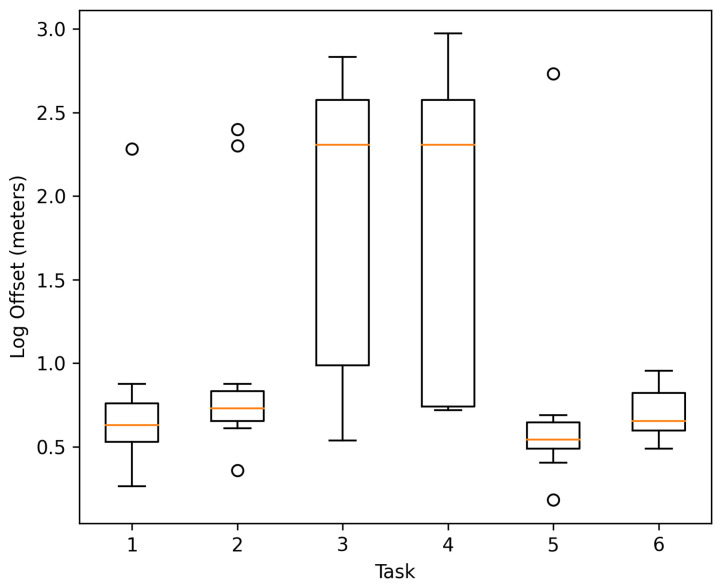
Log distance offset registered by the system while performing the navigation tasks.

During the navigation tasks, the user’s location within the implemented system registered an average offset distance of 3.82 m for the 72 cases, concentrating the most significant offsets in the navigation tasks towards targets 3 and 4, as shown in Figure 6. According to the results, the distance traversed, the environmental illumination, the width of the hallways, and the user’s behavior affected the tracking performed by the motion sensors, causing the major distance offsets in the study.

##### Navigation Time Comparison

**Figure 7:**
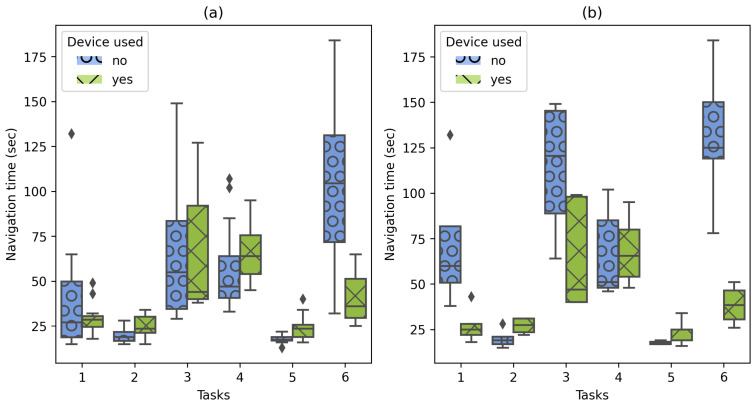
Navigation time recorded with and without the use of the implemented system. (**a**) Time recorded for all cases (144 cases). (**b**) Time recorded in the cases where users were not familiar with the navigation environment (54 cases).

**Figure 8:**
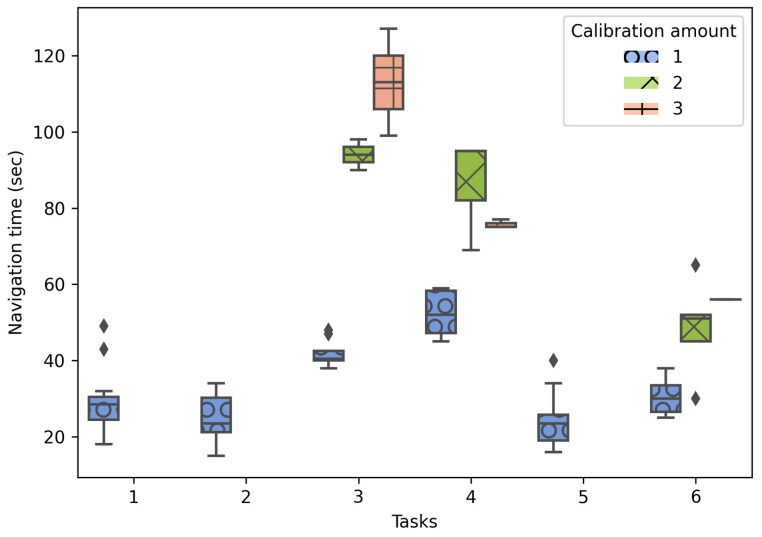
Recorded navigation time considering the amount of calibrations.

The navigation time recorded for the 144 navigation tests produced diverse performance results under various conditions, such as prior knowledge of the environment and unexpected device errors that increased the time to locate a target. As Figure 7 shows, the navigation time results are displayed for the total number of tests (144) in part (a) and for the cases where the user had no prior knowledge of the building in part (b). Please note that the average navigation time to the different targets using the implemented system was only faster on three navigation tasks, being better in situations where the user does not know anything about the destination location. In particular, in situations where the prototype was not used to find the target, users required the assistance of the University’s staff a total of 20 times and deviating on 25 occasions. On the other hand, regarding the QR code scans for repositioning (recalibrations), the users who presented visualization problems of the AR guide (caused by positioning offsets) increased significantly the time required to locate the target. Figure 8 shows the navigation time per task according to the number of required scans. Please note that some tasks did not require recalibrations.

##### AR Display Issues

**Figure 9:**
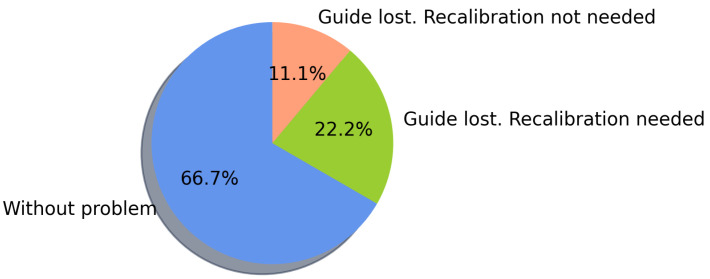
AR line display errors during navigation.

**Figure 10:**
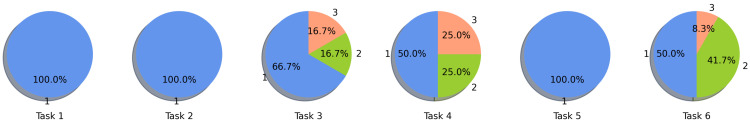
Ratio of QR scans to set position by task. The category values represent the number of QR scans required to complete the navigation.

During the navigation tasks, the user experienced some issues visualizing the AR line to reach the destination. According to Figure 9, within the 72 tests, 56 times (77.7%) the navigation was completed without requiring fixing the position by re-scanning QR codes, and the remaining ones required no more than two repositionings per test. Within these 56 tests where navigation was completed without requiring fixing the position, in 48 situations, the user did not have any visualization problems of the AR line used for guiding, being able to complete the navigation. Thus, the remaining times the user lost visual contact with the AR guide, but only until almost reaching the task’s destination, still being able to finish the navigation. The percentage distribution of the number of QR readings performed to complete the navigation in each task is shown in Figure 10.

##### User Feedback

At the end of the tests, each participant answered a survey related to the general navigation functionality and experiences with the prototype and information consumption from the environment. Based on a five-point Likert agreement scale ranging from “Strongly Disagree” to “Strongly Agree”, the results of the survey (together with the applied questions) are shown in Table 3. Regarding the degree of agreement among participants, the Fleiss’ Kappa coefficient was 43.69%, which is interpreted as a moderate agreement [46], which means the answers were not by chance.

### 5.3. Case 2: Cinvestav Building

This case presents the navigation experiments prepared for a building of Cinvestav Tamaulipas (https://www.tamps.cinvestav.mx, accessed on 7 June 2021) (a research institute in Victoria, Mexico). The idea was to evaluate the feasibility of the proposal under a different scenario (spatial model, mobile device, tasks). The device used to perform the tests was a Google Pixel 4, running Android 10. Due to pandemic restrictions, the developed navigation system was tested slightly differently from the previous case, as described in the following subsections.

#### 5.3.1. Participants

In this navigation environment, five staff members from the institute participated in testing the navigation system. The features of the participants are described as follows:

- The users became familiar with the mobile platform and understood the usefulness and scope of augmented reality applications.
- All participants had prior knowledge of the environment but were instructed and supervised to follow only the directions provided by the device.
- The participants performed the navigation using the same mobile device (alternating its use), generating a dataset of 25 results.

#### 5.3.2. Scenario Description and Navigation Tasks

**Figure 11:**
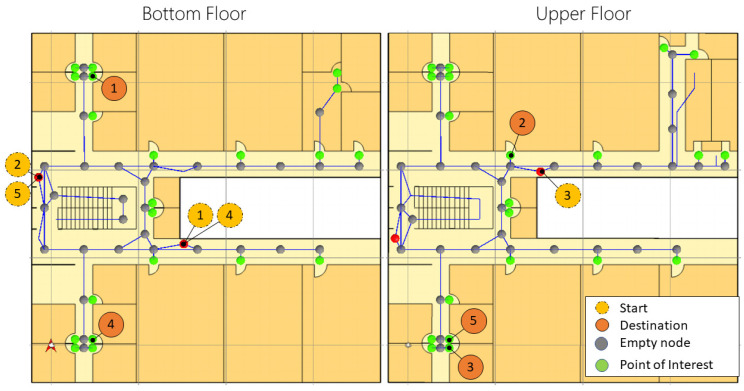
Scenario and navigation tasks for the Cinvestav building.

As acquired for Case 1, this building is composed of two floors, and the plans were digitized to obtain an image of 586 × 586 pixels per floor, obtaining an area of 1600 m^2^. Then, such images were imported into Unity3D under a scale of 14.65 pixels per unit. Regarding the spatial model, 109 nodes and 36 points of interest were defined. The navigation scenario and tasks are depicted in Figure 11, again a QR code is placed on each *Start* point. For this case, due to the current institute’s entry restrictions, five different navigation tasks were established, and every participant performed each of the five tasks, obtaining 25 task records. Table 4 shows the five navigation tasks designed for this scenario.

#### 5.3.3. Results

This section presents the results obtained from the navigation tasks performed within the Cinvestav navigation environment. As previously mentioned, each task was completed five times by the users using the implemented system. Moreover, on average, the time to complete the tasks was 31.84 s, with a traveled distance of 169.39 m per participant. The results for the assessed criteria are described in the following subsections.

##### Position Setting

Within the 25 navigation instances, 35 QR code scans were performed for initial position setting and positioning calibration, with an accuracy of 100% (all scans provided correct positioning).

##### Distance Offset

**Figure 12:**
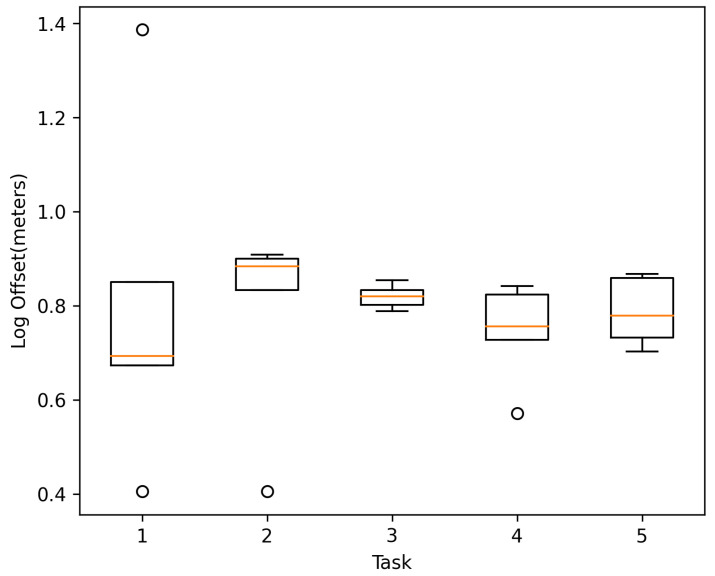
Log distance offset per navigation task.

The continuous tracking of the user’s position during navigation showed good performance, as seen in Figure 12, obtaining an average distance offset of 1.23 m between the user’s final position and the one tracked by the system at the end of navigation.

##### Navigation Time

**Figure 13:**
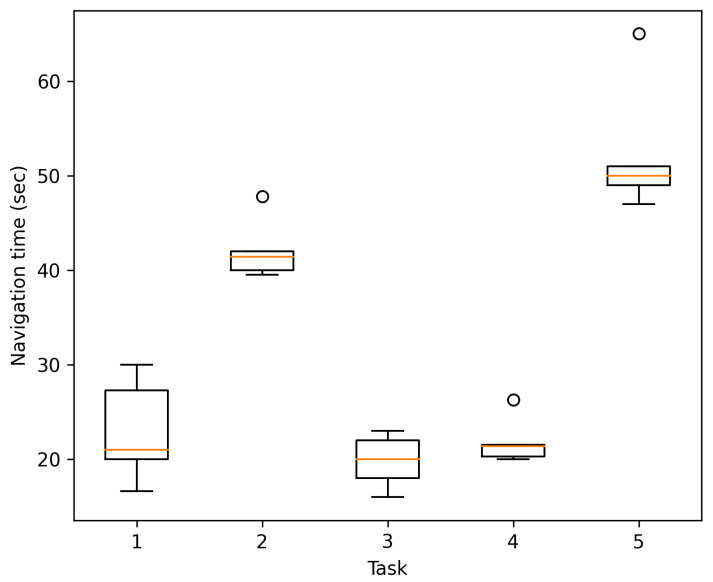
Navigation time per task in the Cinvestav Building.

In this case, all tests consider navigation with the mobile device. In this regard, the navigation time in seconds was recorded to determine the participants’ time to travel the five different routes with varying distances. The navigation time is shown in Figure 13 for all 25 navigation instances (from the five tasks), from the starting QR location to the point of interest. As expected, the navigation time increases as the distance traveled increases, completing any trip in no more than 65 s.

##### AR Display Issues

**Figure 14:**
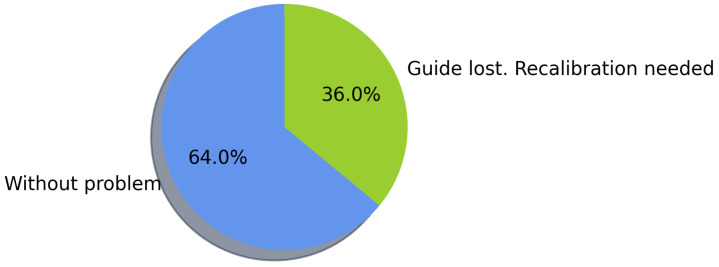
AR line display errors during navigation.

**Figure 15:**
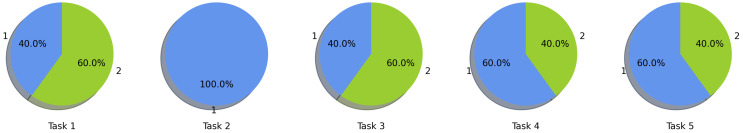
Ratio of QR scans to set position by task. The category values represent the number of times a QR scan was required to complete the navigation.

As shown in Figure 14, within the 25 navigation tasks, in 16 instances, the navigation had no errors (and the participants reached the destination). In contrast, in 9 instances, the guidance was lost, and the system had to be calibrated by scanning a QR code midway to complete the navigation. Among the experiments performed, the navigation to the target in task 2 was the only one that did not present a visualization problem, while the rest of the tasks required at most two QR scans to fix the visualization of the AR guide, as shown in Figure 15.

##### User Feedback

After the navigation tasks, the participants answered a survey about the navigation system’s usefulness and information consumption function. Similar to Case 1, the survey is based on a Likert scale [45]. However, in this case, those questions not involving the navigation system were discarded. The results of the survey are shown in Table 5.

## 6. Discussion

This section discusses the features, results, and limitations presented by the navigation system based on the proposed methodology, according to the evaluation criteria and the prepared navigation scenarios.

### 6.1. Implementation

As previously mentioned, the implemented prototype is based on a mobile application system whose main components are the AR for content display and the Semantic Web for data management. Even though other tools can be incorporated into the prototype, the purpose of each module in the methodology is fulfilled without incorporating external elements for positioning or sensing. However, different tools will be considered for a future version of the system.

### 6.2. Position Setting

The resulting performance for establishing the initial position by scanning QR codes is primarily positive, obtaining an accuracy close to 100% in both cases where the prototype was tested. In this sense, there were issues on three instances in total, positioning the user in the wrong coordinates inside the system and causing to display the AR elements in the wrong location, as their position should be relative to the user’s initial location. In these cases, when performing a QR code scan, the system registered several changes in the detected feature points falsely, causing such initial positioning to be incorrect. The main reason for this issue is how AR works to establish and track the user’s positioning, being dependent on the environment’s characteristic features (e.g., object’s edges and textures) to register the user’s movement [47]. These feature points are obtained through algorithms (SIFT, FAST, BRISK) that process the pixels of the captured images under conditions such as color differences in the pixels around a central point, being affected by the illumination and the monotony of the environment. The current solution to this issue was to re-scan the QR code to fix the location.

### 6.3. Distance Offset

**Figure 16:**
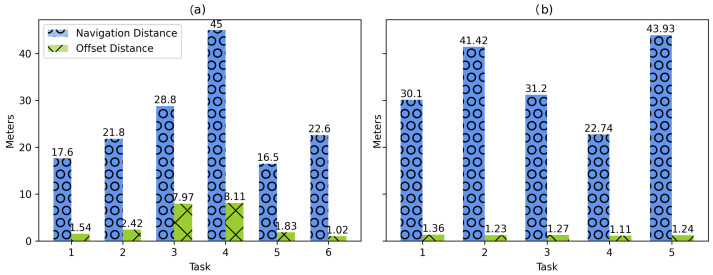
Navigation distance vs. offset distance by task. (**a**) University distances, (**b**) Cinvestav distances.

**Figure 17:**
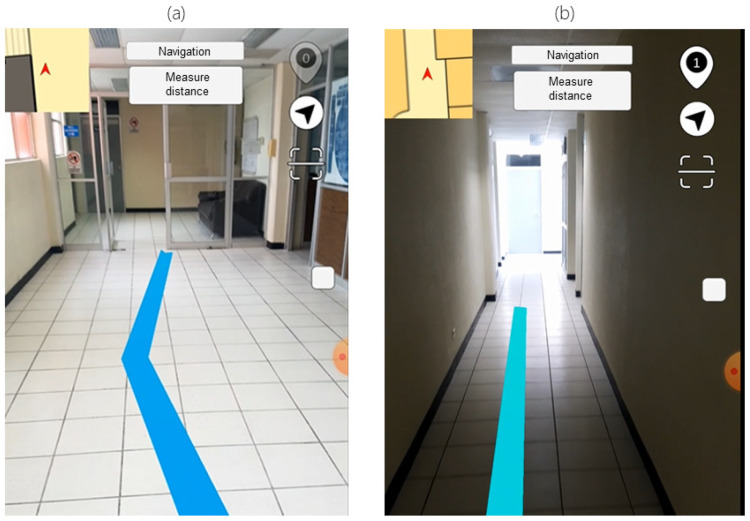
Comparison of routes. (**a**) Route with good illumination and environmental characteristics. (**b**) Route with low illumination and few environmental characteristics.

During the experiments, the system’s distance offset was recorded to measure the tracking functionality. According to the results obtained, the navigation to targets 3 and 4 in Case 1 presented the largest distance offsets, affecting the visualization of the AR content. Although it was initially assumed that there is a relationship between navigation distance and offset distance, Figure 16 shows that this is not the case. Thus, navigation to points of interest with relatively long distances presented similar offsets to other short-distance routes (also presented in results of Case 2). In this sense, illumination, shadows, and difference in textures are reasons that negatively impact computer vision-aided positioning and tracking systems [48]. Therefore, it is necessary to have a good level of illumination in the corridors and to avoid pointing the camera of the mobile device at walls or objects of monotonous colors to be able to detect enough environmental characteristic features within the environment. Such issue is shown in Figure 17.

### 6.4. Navigation Time

For Case 1, a navigation time comparison is presented for participants using the navigation system (or not) for the six navigation tasks. The results demonstrated faster navigation times on four out of the six tasks when the system is used. In this regard, the navigation time is mainly affected by factors such as prior knowledge of the environment, or the AR guide display issues encountered during the experiment as shown in Figure 7 and Figure 8. For example, in task 6, when the system is not used, the point of interest does not contain any landmark to help identify the destination, which causes the users to request support from people to find the navigation target and, in this way, justifying the development of this research work. In the cases where the system was slower than navigation without its use, it was because the location of the targets was relatively simple (there were signs indicating their location) or the users knew the location beforehand, allowing them to navigate with more confidence and faster.

On the other hand, although Case 2 does not demonstrate the time with those participants not using the navigation system, it was possible to collect the navigation time, offset distances, and positioning scans to test the feasibility of the proposed method. However, even though the testing conditions were different for both cases (e.g., tasks, participants, scenario dimensions, corridor widths), the participant speed results indicated a median of 0.63 m/s and 1.03 m/s for Cases 1 and 2, respectively. Such speed results provide an initial idea of the system’s behavior for further fine-grained evaluations on general indoor navigation systems with varying participant ages, physical limitations, and possible routes, to mention a few.

### 6.5. AR Display Issues

Regarding the AR content presentation, on average, 70.9% of the navigation tasks for both implementation cases did not present any visualization problem regarding the augmented objects used to present the navigation route, therefore not needing to calibrate the system to complete the navigation (only required for the initial positioning). As for those that presented visualization problems, the route presented on the screen did not match the corridors of the building, thus confusing the user. These presented visualization issues were mainly due to the position tracking and distance offset, generated by situations previously discussed regarding feature points detected from the environment, thus requiring a system calibration. Since a positioning calibration may be required anywhere in the building to restore the correct position, it was decided to place the QR codes in the main locations of the buildings, covering a radius of 7 m per code.

### 6.6. User Feedback

As part of the navigation tests, 27 different people shared their opinion by answering a survey. In general, most users expressed a favorable impression of the system, considering the AR interactive enough for indoor navigation and finding helpful information provided by the KB. In Case 1, since the University currently lacks a database for the extraction of contact information of the academic staff, users appreciate being able to obtain such information at hand and even know the research and teaching activities they perform, as it benefits them when seeking advisor for the development of research projects. Overall, their opinion is affected according to the experience obtained using the application, being more favorable in those cases where users presented minor AR display difficulties or locating an unfamiliar point of interest. For example, new users find the system very useful to get to know the institution and their professors better, especially when looking for someone in particular. On the other hand, users in higher grades see the availability of the information provided by the system as favorable but consider that they can navigate faster within the environment without the help of the system. Even so, several of these users mentioned that they were unfamiliar with many of the professors and their offices despite having been at the institution for four years.

### 6.7. Data Model

Although existing navigation works use traditional database solutions, a KB supported by an ontology provides semantic and structural advantages for managing the data in navigation environments with varying features. For example, due to the different roles of teachers and staff in the academic domain, modeling data can be complex with a relational database, requiring join and union operators to check the availability of components (in the case of null values), which can complicate the definition of queries [49]. Thus, the ontology provides the properties to define environment features, and the contextual information can be consulted more directly (as presented in Listing 1). Otherwise, the definition of multiple tables and foreign keys would be required. Moreover, using a KB provides a specialization of the points of interest that allow the disambiguation of entities and definition of hierarchies (e.g., a classroom part of a department within a floor). For example, if there is a laboratory called “Alan Turing” but a professor has the same name, the ontology allows differentiation of both entities according to their classes. On the other hand, there might be entities with different names (synonyms), but the same meaning supported by the KB. For example, “Lab-1” is also known as “Alan Turing” laboratory. Another advantage is that the KB follows a platform-independent standardization useful for data sharing and consumption [32]. Although the inference process has not yet been exploited for this work, rules could be generated to take advantage of the KBs in a further system version.

### 6.8. Data Storage and Consumption

**Figure 18:**
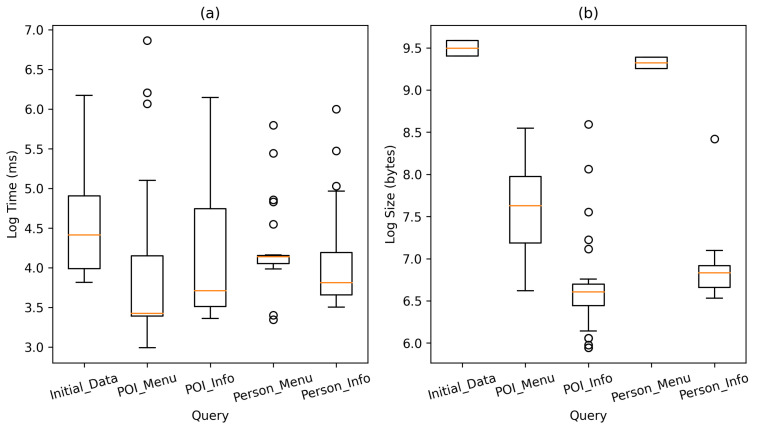
(**a**) Average time in milliseconds per query, (**b**) Average size in bytes per query.

Although several RDF data stores can be used to implement the proposed methodology, Virtuoso was initially chosen because of its free version and popularity in projects such as DBpedia [31]. It also fulfills the necessary tasks to store the KB and serve queries in SPARQL. On the other hand, various studies show competitive results for Virtuoso in different tests of throughput, query latency, and query response time regarding existing RDF stores [50,51,52]. However, to capture the prototype information consumption performance, an analysis of the response time and size was conducted between the mobile device and the server. In this sense, the five SPARQL queries mentioned in Section 4.2 were submitted to the Virtuoso server inside a local wireless network. Each query was submitted 31 times to get an average performance using random entities regarding points of interest and persons. Thus, the number of returned RDF triples was varied for each query response. The results of the response time and size are shown in Figure 18 on a logarithmic scale. The implementation shows a response time in the range of 18 to 965 ms, with an average of 100 ms. In general, the query that collects the initial data for the system presents the highest average response time compared to the other queries (144 ms). On the other hand, the size of the responses is strongly linked to the number of triples extracted from the server, presenting different sizes among the same type of query because the points of interest and persons in the KB contain different amounts of information. The response sizes varied from 0.381 Kb to 14.559 Kb, with the initial data query again presented the largest response size with an average of 13.35 Kb.

### 6.9. Limitations

Due to the proposed use of AR as a positioning method, it is necessary to have a device compatible with the AR framework [53]. In this sense, the system is limited to illumination conditions and extraction of characteristic points in the environment for its correct operation. In addition, this technology makes intensive use of the processor’s device camera, causing the device’s battery to be drained quickly [54]. At the same time, due to the proposed client-server model handling the KB, the system requires a server connection to obtain the points of interest of the environment and its information.

### 6.10. Application Usage

The availability of an indoor navigation system provides advantages in several situations, giving more independence to the user while looking for specific locations in unfamiliar environments. In indoor scenarios, there are several situations in which navigation without a system can become more difficult for users. For example, when there are not enough signs to guide users through the corridors, the provided maps are not up to date, there are no people available around to provide clear directions, and so on. Even though some errors occurred when displaying the virtual navigation guide in the system, the user had the option to recalibrate its position and then reach its destination. Thus, through the implemented system, the search for indoor locations becomes a more independent activity, providing contextual and updated information on the locations of places by updating the nodes and linking them to the KB. In this regard, one of the contributions of the methodology is to provide the information of points of interest through the KB following Semantic Web standards, which offers the option to consume such information independently of the platform by users and applications.

## 7. Conclusions

This paper presented a novel methodology for developing indoor navigation systems, integrating AR and Semantic Web technologies to visualize routes and consume contextual information of the environment. Based on the design of the proposal, it was developed a prototype system implementation for testing purposes. The system was tested on two cases of study into academic environments, designing and implementing an ontology (and a KB) for supporting the information in such a domain. Moreover, it was modeled the information of plans, routes, locations, and points of interest for each case of study. According to the results obtained from the navigation experiments, the AR (and computer vision) effectiveness as the positioning method is determined, being highly dependent on detecting environmental characteristics and illumination conditions of the environment for better tracking user’s movements. The positive feedback received from the users who participated in the experimentation and the response of the system on the two modeled scenarios demonstrated the feasibility of the proposed methodology, resulting in easier and faster navigation to points of interest whose location was unknown to the users. As future work, we wish to explore other methods for position tracking (such as WiFi or Bluetooth wireless networks) to reduce location problems using the currently available infrastructure. In addition, it is intended to take further advantage of AR and Semantic Web to offer a greater variety of ways to present contextual information while navigating without cluttering the user interface, even in other contextual environments aside from academic institutions.

## Figures and Tables

**Table 1:** Indoor navigation system components. Some options refer to Graph Model (GM) or those Not Implemented (–).

Work	Components					
	Spatial Model	Position Tracking	Data Storing	Pathfinding Algorithm	Navigation Content Display	POI Information Content
Al Delail et al. [23]	Third-party map	Marker detection and Mobile’s sensors	MySQL	A*	Lines on a digital plane	Location name
INSAR [6]	GM	WiFi fingerprinting, compass	SQLite	Record matching	AR arrows	–
Matuszka et al. [16]	Third-party map	Marker detection and Mobile’s sensors	Semantic Web	No info	AR arrows and Lines on a digital plane	–
Cankaya et al. [20]	GM	Mobile’s sensors	Database (Not specified)	Dijkstra	AR arrows	–
Noreikis et al. [28]	GM	Features matching, camera	Not specified	A*	AR arrows	–
Gang and Pyun [3]	3D model	Bluetooth, Geomagnetic field, Marker detection and Mobile’s sensors	Database (Not specified)	Dijkstra	AR lines	–
Huang et al. [21]	3D model	Lbeacons	No info	Dijkstra	AR arrows	–
Kumar et al. [15]	3D model	Marker detection	Firebase	A*	AR arrows	–
Ng and Lim [27]	GM	WiFi fingerprinting, sensors	SQLite	No info	Robot guide	Info at search
Chidsin et al. [24]	Point cloud	RGB-D camera	No info	SLAM	AR arrows	Location name

**Table 2:** University building navigation tasks: *BF* denotes Bottom Floor, and *UF* denotes Upper Floor.

Start Point	Start Floor	Destination	Destination Floor	Distance (Meters)
1	*BF*	Laboratory 1	*BF*	17.60
2	*BF*	Men bathroom	*BF*	21.80
3	*BF*	Coordination Office	*UF*	28.80
4	*UF*	Classroom 5	*UF*	45.00
5	*UF*	Reception	*BF*	16.50
6	*UF*	Researcher Office	*UF*	22.60

**Table 3:** Average result of the survey applied on a Likert scale. 1 = Strongly disagree, 2 = Disagree, 3 = No preference, 4 = Agree, 5 = Strongly agree.

Question	Average
1. Navigation with the application was easier than navigation without its use.	4
2. Navigation time with the application was faster than without its use.	4
3. The application gives you more confidence in finding locations within a building.	5
4. The points of interest information provided by the application is useful in a school setting.	4
5. The teacher information provided by the application is useful in a school setting.	5
6. The included mini-map is a navigational aid.	4
7. The assistance of the application to find places is preferred rather than asking for directions.	4

**Table 4:** Navigation tasks for Cinvestav’s building: *BF* denotes Bottom Floor, and *UF* denotes Upper Floor.

Start Point	Start Floor	Destination	Destination Floor	Distance (Meters)
1	*BF*	Office of Profr. 1	*BF*	30.10
2	*BF*	Laboratory 8	*UF*	41.42
3	*UF*	Office of Prof. 2	*BF*	31.20
4	*BF*	Office of Prof. 3	*BF*	22.74
5	*BF*	Office of Prof. 2	*UF*	43.93

**Table 5:** Average result of the survey applied on a Likert scale. 1 = Strongly disagree, 2 = Disagree, 3 = No preference, 4 = Agree, 5 = Strongly agree.

Question	Average
1. The application gives you more confidence in finding locations within a building	5
2. The points of interest information provided by the application is useful in a school setting	5
3. The teacher information provided by the application is useful in a school setting.	5
4. The included mini-map is a navigational aid.	4
5. The assistance of the application to find places is preferred rather than asking for directions.	5

## Data Availability

Not applicable.

## Abbreviations

The following abbreviations are used in this manuscript:

AR	Augmented Reality
KB	Knowledge base
